# Supplementary material for: The impact of flipping class intervention on reading comprehension: Different approaches and proficiency levels
Source: PLoS One. 2024 Jun 20;19(6):e0305041. doi: 10.1371/journal.pone.0305041 (PMC11189174; doi:10.1371/journal.pone.0305041)
Supplement: Attachment — Submitted filename: PONE-D-23-42134 Comments.docx [file pone.0305041.s001.docx]

PONE-D-23-42134

Overall comments:

This study "The Impact of Flipping Class Intervention on Reading Comprehension: Different Approaches and Proficiency Levels" examines an important educational strategy. However, the methodology presents challenges in participant selection and assignment, which could influence the study's validity due to potential biases or lack of representativeness. The generalizability of findings to a wider educational context is not clearly established.

Another big question is that the theoretical framework needs more comprehensive integration with the empirical work, and the analysis does not fully address potential confounding variables. There might be a need for more rigorous statistical testing to strengthen the evidence for the claimed effects in the discussion part. Also, it might be important to also discuss the flipped classroom model's application across diverse educational settings.

Introduction:

1. **Row 1, Page 1**: While the introduction establishes the relevance of flipped classrooms in language learning, it lacks a direct comparison with traditional methods. Suggestion: Briefly contrast flipped classrooms with conventional teaching approaches to highlight specific benefits or challenges.
2. **Row 6, Page 1**: The claim that flipped classes improve reading outcomes in struggling readers needs further substantiation. Suggestion: Clarify why this model is particularly effective for these students.
3. **Row 12, Page 1**: The discussion on engaging materials in flipped classes is good but lacks a comparative aspect. Suggestion: Contrast with traditional methods using similar materials to identify unique benefits of the flipped approach.
4. **Row 18, Page 1**: The introduction points out potential failures in flipped classroom implementations but needs more specificity. Suggestion: Elaborate on what constitutes effective vs. ineffective design in flipped classrooms.
5. **Row 22, Page 1**: Technology and internet access are noted as prerequisites but without solutions for challenges. Suggestion: Briefly mention potential strategies to overcome technological barriers, especially in resource-limited settings.
6. **Row 27, Page 2**: The research question is clear but lacks contextual depth. Suggestion: Explicitly state how this study adds new dimensions to existing research on flipped classrooms in language learning.

Literature review:

1. **Schema Activation (Page 3)**: The authors discuss schema activation in reading comprehension, referencing various studies. It's suggested that the manuscript elaborates on how these studies directly relate to the specific context of flipped classroom interventions. This would strengthen the connection between general theory and the study's specific focus.
2. **Mixed Study Findings (Page 3)**: The literature review presents conflicting findings regarding the use of technology in promoting schema activation. While this is valuable, the authors should offer a more critical analysis of why these discrepancies exist and what implications they hold for the current study.
3. **Flipped Classroom Studies (Page 5)**: The review cites several studies supporting flipped classroom efficacy but seems to lack a discussion on the methodological strengths and weaknesses of these studies. Providing such an analysis would offer a more nuanced understanding of the flipped classroom's potential benefits and limitations.
4. **Video-Based Pre-Reading (Pages 12-13)**: The discussion on video-based pre-reading is insightful, yet it could be enhanced by a more detailed exploration of how these strategies specifically impact reading comprehension in the flipped classroom context, considering factors like student engagement and cognitive load.
5. **Effectiveness of Flipped Classroom Strategies (Page 17)**: While the review discusses the varying effectiveness of flipped classroom strategies, it would benefit from a deeper examination of the factors influencing these outcomes, such as student characteristics, instructional design, and the nature of reading materials used.

Method:

1. **Research Design**: The quasi-experimental design due to the non-random assignment of participants is acknowledged. However, the lack of randomization could introduce selection bias affecting the study's internal validity. The similarities between the participant groups from two different universities are noted, yet the study could benefit from a more detailed explanation of how these similarities were assessed and ensured.
2. **Ethical Considerations**: The study's ethical approval and informed consent procedures are well documented, demonstrating adherence to ethical standards. This is an important aspect, as it assures the ethical integrity of the research.
3. **Intervention Formats**: The manuscript describes two formats of flipped class intervention (A and B) with detailed descriptions of pre-reading, while-reading, and post-reading activities. While this thorough description is commendable, it would be beneficial to include a rationale for the selection of these specific activities and their expected impact on reading comprehension.
4. **Pilot Test**: The conduct of a pilot test is a strong point, helping to validate the research procedures and assessment tools. However, more information on how the pilot test findings were used to refine the study would be useful.
5. **Data Collection Tools**: The use of the TOEFL ITP® Assessment Series to gauge reading competency is appropriate, but there's a lack of detail on how these tests align with the specific objectives of the study. Additionally, including qualitative data through interviews enriches the study, yet the selection criteria for interview participants and the method of analysis should be more explicitly detailed.
6. **Statistical Analysis**: The use of non-parametric statistical methods due to the small sample size is appropriate. The manuscript provides a clear explanation of the statistical tests used and the rationale behind their selection. However, a discussion on the limitations of these methods given the sample size and study design would strengthen this section.
7. **Sample Size and Generalizability**: The small sample size is acknowledged as a limitation. This impacts the generalizability of the findings, and the study would benefit from a discussion on how these limitations could affect the interpretation of the results.

Discussion:

1. **Clarity and Precision of Results**: The manuscript presents clear findings comparing the efficacy of different flipped classroom formats (A and B) and their impact on students with varying proficiency levels. However, there seems to be a lack of depth in the statistical analysis. For example, while the median scores and P-values are mentioned, there is no discussion on effect sizes or confidence intervals, which are crucial for understanding the practical significance of the findings.
2. **Discussion and Interpretation**: The authors have linked their findings to existing literature, acknowledging both the supportive and opposing views regarding the effectiveness of flipped classroom interventions. This balanced approach is commendable as it provides a comprehensive understanding of the subject. However, the discussion could be enhanced by delving deeper into why certain strategies were more effective, possibly exploring learner characteristics, instructional design, or the nature of reading materials used.
3. **Lack of Critical Examination of Methodological Limitations**: While the authors briefly acknowledge the quasi-experimental design, there is insufficient critical reflection on how this and other methodological choices might have influenced the results. For instance, the non-random assignment of participants to groups could introduce selection bias, affecting the generalizability of the findings.
4. **Implications for Practice**: The manuscript offers useful insights for educators and instructional designers, especially regarding the importance of tailoring interventions to learners' proficiency levels. However, the practical implications could be more explicit. For instance, how can educators in similar contexts apply these findings? What are the specific strategies that proved effective in Format B, and how can they be replicated or adapted in different educational settings?
5. **Suggestions for Future Research**: The authors could strengthen the conclusion by suggesting specific directions for future research. This could include exploring the long-term impact of flipped classroom interventions on reading comprehension, examining how these approaches work for different age groups, or investigating the role of learner motivation and self-regulation in such interventions.
