# Supplementary material for: The impact of flipping class intervention on reading comprehension: Different approaches and proficiency levels
Source: PLoS One. 2024 Jun 20;19(6):e0305041. doi: 10.1371/journal.pone.0305041 (PMC11189174; doi:10.1371/journal.pone.0305041)
Supplement: Attachment — Submitted filename: Rebuttal letter.docx [file pone.0305041.s002.docx]

**Rebuttal letter**

PONE-D-23-42134

Overall comments:

This study "The Impact of Flipping Class Intervention on Reading Comprehension: Different Approaches and Proficiency Levels" examines an important educational strategy. However, the methodology presents challenges in participant selection and assignment, which could influence the study's validity due to potential biases or lack of representativeness. The generalizability of findings to a wider educational context is not clearly established.

**My response:**

Thanks for the feedback.

Your concerns regarding participant selection and assignment in the methodology are valid and merit attention. The potential biases or lack of representativeness could indeed impact the study's validity. To address these challenges, future research might consider employing more rigorous participant selection methods, such as randomization, to enhance the study's internal validity.

Another big question is that the theoretical framework needs more comprehensive integration with the empirical work, and the analysis does not fully address potential confounding variables. There might be a need for more rigorous statistical testing to strengthen the evidence for the claimed effects in the discussion part. Also, it might be important to also discuss the flipped classroom model's application across diverse educational settings.

**My response:**

Thank you for your insightful feedback. While we recognize the importance of participant selection and assignment in ensuring study validity, we encountered resource constraints, particularly in terms of time and personnel, which limited our ability to implement additional analyses and perform more rigorous statistical testing. These limitations stem from the initial scope and objectives of our study, which were carefully designed to address specific research questions within the available resources. Furthermore, we acknowledge the importance of addressing potential confounding variables and providing a more comprehensive integration of the theoretical framework with the empirical work. However, due to the constraints mentioned earlier, implementing these changes post-study completion poses challenges. In terms of the generalizability of findings, we acknowledge that our study focuses on a specific educational context, and while we aimed to draw meaningful insights, extending the results to a broader educational context was not a primary objective of our research. We appreciate your understanding of these constraints and hope that the current manuscript, within its defined scope and limitations, contributes valuable insights to the academic community.

Introduction:

1. **Row 1, Page 1**: While the introduction establishes the relevance of flipped classrooms in language learning, it lacks a direct comparison with traditional methods. Suggestion: Briefly contrast flipped classrooms with conventional teaching approaches to highlight specific benefits or challenges.

**My response:** I have addressed the comment by including a brief contrast between flipped classrooms and conventional teaching approaches to highlight specific benefits and challenges.

2. **Row 6, Page 1**: The claim that flipped classes improve reading outcomes in struggling readers needs further substantiation. Suggestion: Clarify why this model is particularly effective for these students.

**My response:** Thank you for your feedback. I've revised the article to address your comment. I emphasized that struggling readers benefit from the extra time provided by flipped learning, allowing them to access materials at their own pace

3. **Row 12, Page 1**: The discussion on engaging materials in flipped classes is good but lacks a comparative aspect. Suggestion: Contrast with traditional methods using similar materials to identify unique benefits of the flipped approach.

**My response**: I've made the changes you suggested to the article. Now, I've compared flipped classes with traditional methods. In the revised paragraph, I pointed out that flipped classes have a unique advantage in engaging and motivating students. I highlighted the interactive online resources they use, like games, simulations, virtual labs, and films.

4. **Row 18, Page 1**: The introduction points out potential failures in flipped classroom implementations but needs more specificity. Suggestion: Elaborate on what constitutes effective vs. ineffective design in flipped classrooms.

**My response:** I've made the changes you suggested to the introduction. I mentioned that the success of flipped class programs depends on how well they're designed and carried out. If poorly structured, these programs can face issues. This includes trouble with navigation, unclear instructions, a lack of interactive elements, or content that's not suitable for the students' age.

5. **Row 22, Page 1**: Technology and internet access are noted as prerequisites but without solutions for challenges. Suggestion: Briefly mention potential strategies to overcome technological barriers, especially in resource-limited settings.

**My response**: I've updated the article based on your suggestion. I added some ideas to tackle the tech and internet challenges, especially in places with fewer resources. I mentioned things like using low-data ways to share content, using stuff offline when you can, and suggesting community partnerships for more tech access. These ideas aim to make sure flipped learning works well even in spots with limited tech resources

6. **Row 27, Page 2**: The research question is clear but lacks contextual depth. Suggestion: Explicitly state how this study adds new dimensions to existing research on flipped classrooms in language learning.

**My response:**

I've updated the article based on your suggestion. Now, I've explained how our study fits into the bigger picture of flipped classroom research. We compare our findings with other studies to see if there are patterns or differences and to figure out what factors might be causing different outcomes. This helps us give a full picture of how flipped classrooms work in language learning, especially concerning reading comprehension for Indonesian university students.

Literature review:

1. **Schema Activation (Page 3)**: The authors discuss schema activation in reading comprehension, referencing various studies. It's suggested that the manuscript elaborates on how these studies directly relate to the specific context of flipped classroom interventions. This would strengthen the connection between general theory and the study's specific focus.

**My response**:

I wanted to let you know that I've made revisions to the article, incorporating your suggestion to connect the discussion of schema activation with the specific context of flipped classroom interventions. I highlighted how flipped classrooms, through multimedia resources, interactive activities, and collaborative virtual environments, serve as effective tools in promoting schema activation for improved reading comprehension.

2. **Mixed Study Findings (Page 3)**: The literature review presents conflicting findings regarding the use of technology in promoting schema activation. While this is valuable, the authors should offer a more critical analysis of why these discrepancies exist and what implications they hold for the current study.

**My response:**

I've revised the article based on your feedback. I concluded that the impact of technology on schema activation is intricate. The conclusion suggests that understanding the complexity and various influences on schema activation due to technology underscores the importance of customized teaching methods, digital literacy, well-thought-out online learning environments, balancing the pros and cons of technology, and ongoing research to improve educational practices.

3. **Flipped Classroom Studies (Page 5)**: The review cites several studies supporting flipped classroom efficacy but seems to lack a discussion on the methodological strengths and weaknesses of these studies. Providing such an analysis would offer a more nuanced understanding of the flipped classroom's potential benefits and limitations.

**My response**:

I revised the section on flipped classrooms based on your feedback:

1. One study (32) focused on 30 higher education students, offering in-depth analysis but with limited generalizability to other educational levels.

2. Another study (33) examined 120 fourth-grade students, providing insights into K-12 settings but posing limitations for higher education and age-related considerations.

3. A third study (21) involved 63 non-native English speakers, adding diversity and relevance to language learning but potentially lacking generalizability to native speakers. Common considerations include small sample sizes, varied intervention durations, and different measures of reading comprehension, impacting result comparability. While valuable, cautious interpretation is needed due to nuanced methodological aspects related to participant demographics, settings, and measurement tools.

4. **Video-Based Pre-Reading (Pages 12-13)**: The discussion on video-based pre-reading is insightful, yet it could be enhanced by a more detailed exploration of how these strategies specifically impact reading comprehension in the flipped classroom context, considering factors like student engagement and cognitive load.

**My response**:

I've made revision. The videos sum up key concepts, making learning more engaging and relevant, potentially improving memory. In this personalized learning setup, students go at their own pace, avoiding cognitive overload and deepening understanding.

5. **Effectiveness of Flipped Classroom Strategies (Page 17)**: While the review discusses the varying effectiveness of flipped classroom strategies, it would benefit from a deeper examination of the factors influencing these outcomes, such as student characteristics, instructional design, and the nature of reading materials used.

**My response**:

Thank you for your valuable feedback. I've taken your suggestion to heart and expanded the review to delve deeper into the factors influencing the effectiveness of flipped classroom strategies, including student characteristics, instructional design, and the nature of reading materials. I agree that further studies could provide a more in-depth exploration of these aspects to enhance our understanding of the nuanced dynamics at play. If you have any additional insights or specific areas you'd like me to emphasize, please feel free to let me know.

Method:

1. **Research Design**: The quasi-experimental design due to the non-random assignment of participants is acknowledged. However, the lack of randomization could introduce selection bias affecting the study's internal validity. The similarities between the participant groups from two different universities are noted, yet the study could benefit from a more detailed explanation of how these similarities were assessed and ensured.

**My response**:

Thank you for your feedback. We ensured participant similarities by setting specific criteria during selection, including first-year students from both universities in non-English departments, attending English as a general course, and demonstrating intermediate English proficiency. The influence of university administrators in scheduling for all students standardized time constraints, enhancing the study's internal validity.

2. **Ethical Considerations**: The study's ethical approval and informed consent procedures are well documented, demonstrating adherence to ethical standards. This is an important aspect, as it assures the ethical integrity of the research.

**My response**:

Thank you

3. **Intervention Formats**: The manuscript describes two formats of flipped class intervention (A and B) with detailed descriptions of pre-reading, while-reading, and post-reading activities. While this thorough description is commendable, it would be beneficial to include a rationale for the selection of these specific activities and their expected impact on reading comprehension.

**My response**:

Thank you for the feedback. I have given the rationale for activities in formats A and B. These strategies aim to optimize reading comprehension through a mix of in-person and online elements.

4. **Pilot Test**: The conduct of a pilot test is a strong point, helping to validate the research procedures and assessment tools. However, more information on how the pilot test findings were used to refine the study would be useful.

**My response**:

Thank you for your feedback. Based on the findings from the pilot test, we have made necessary adjustments to improve the overall design of the study.

5. **Data Collection Tools**: The use of the TOEFL ITP® Assessment Series to gauge reading competency is appropriate, but there's a lack of detail on how these tests align with the specific objectives of the study. Additionally, including qualitative data through interviews enriches the study, yet the selection criteria for interview participants and the method of analysis should be more explicitly detailed.

**My response**:

Thank you for the feedback. The deliberate choice of the TOEFL ITP® Assessment Series aligns closely with our study's objectives, assessing skills integral to flipped class outcomes. The mapping onto the Common European Framework of Reference ensures effective categorization of reading proficiency levels. Next, the selection criteria for interview participants have been expanded to ensure a comprehensive representation from both instructional methods (A and B).

6. **Statistical Analysis**: The use of non-parametric statistical methods due to the small sample size is appropriate. The manuscript provides a clear explanation of the statistical tests used and the rationale behind their selection. However, a discussion on the limitations of these methods given the sample size and study design would strengthen this section.

**My response**:

Thank you for your feedback. We appreciate your insights. The use of non-parametric statistical methods, driven by our small sample size, indeed poses limitations on generalizability. The choice of the Mann-Whitney test and independent Sample t-Test introduces constraints, primarily due to non-normality assumptions and small sample sizes. We acknowledge these limitations and caution against broad generalization. Future research with larger, diverse samples will enhance the robustness and applicability of our findings.

7. **Sample Size and Generalizability**: The small sample size is acknowledged as a limitation. This impacts the generalizability of the findings, and the study would benefit from a discussion on how these limitations could affect the interpretation of the results.

**My response**:

We have addressed this issue.

Discussion:

1. **Clarity and Precision of Results**: The manuscript presents clear findings comparing the efficacy of different flipped classroom formats (A and B) and their impact on students with varying proficiency levels. However, there seems to be a lack of depth in the statistical analysis. For example, while the median scores and P-values are mentioned, there is no discussion on effect sizes or confidence intervals, which are crucial for understanding the practical significance of the findings.

**My response**:

Thank you for highlighting the importance of discussing effect sizes and confidence intervals. In response, I have revised the manuscript to include a more in-depth statistical analysis, considering effect sizes and confidence intervals to enhance the interpretation of findings.

2. **Discussion and Interpretation**: The authors have linked their findings to existing literature, acknowledging both the supportive and opposing views regarding the effectiveness of flipped classroom interventions. This balanced approach is commendable as it provides a comprehensive understanding of the subject. However, the discussion could be enhanced by delving deeper into why certain strategies were more effective, possibly exploring learner characteristics, instructional design, or the nature of reading materials used.

**My response**:

Thanks for your suggestions. I have made some changes.

3. **Lack of Critical Examination of Methodological Limitations**: While the authors briefly acknowledge the quasi-experimental design, there is insufficient critical reflection on how this and other methodological choices might have influenced the results. For instance, the non-random assignment of participants to groups could introduce selection bias, affecting the generalizability of the findings.

**My response**:

Thanks for the feedback.We acknowledge its limitations, particularly the potential for selection bias due to the non-random assignment, which may impact the generalizability of the findings.

4. **Implications for Practice**: The manuscript offers useful insights for educators and instructional designers, especially regarding the importance of tailoring interventions to learners' proficiency levels. However, the practical implications could be more explicit. For instance, how can educators in similar contexts apply these findings? What are the specific strategies that proved effective in Format B, and how can they be replicated or adapted in different educational settings?

**My response**:

Thank you for highlighting the need for more explicit practical implications. In response, I have provided specific strategies from Format B that educators can adopt in similar contexts, focusing on video-based pre-reading activities and personalized learning experiences. These strategies can be adapted and replicated by introducing engaging videos, exploring personalized learning platforms, offering teacher training, and ensuring continuous evaluation. This ensures a nuanced and evidence-based approach to enhance reading skills across diverse educational settings.

5. **Suggestions for Future Research**: The authors could strengthen the conclusion by suggesting specific directions for future research. This could include exploring the long-term impact of flipped classroom interventions on reading comprehension, examining how these approaches work for different age groups, or investigating the role of learner motivation and self-regulation in such interventions.

**My response**:

Thank you for your feedback. I have added some information about future research directions. Researchers could explore the long-term effects of flipped classroom interventions on reading comprehension, assess applicability across different age groups, and delve into the influence of learner motivation and self-regulation within these interventions.
