# Supplementary material for: The impact of flipping class intervention on reading comprehension: Different approaches and proficiency levels
Source: PLoS One. 2024 Jun 20;19(6):e0305041. doi: 10.1371/journal.pone.0305041 (PMC11189174; doi:10.1371/journal.pone.0305041)
Supplement: Attachment — Submitted filename: Response to Reviewers.docx [file pone.0305041.s003.docx]

**'Response to Reviewers'.**

**Comments to the Author**

1. If the authors have adequately addressed your comments raised in a previous round of review and you feel that this manuscript is now acceptable for publication, you may indicate that here to bypass the “Comments to the Author” section, enter your conflict of interest statement in the “Confidential to Editor” section, and submit your "Accept" recommendation.

**Reviewer #2: (No Response):**

**Our response:** Dear Reviewer #2,

We deeply appreciate your ongoing involvement with our manuscript. Your meticulous review of our revisions has been invaluable. Taking into account your insights, we've meticulously addressed every point from the previous review round. Significant enhancements have been made across various sections including methodology, introduction, literature review, method, discussion, and conclusion, with the aim of improving both the quality and clarity of our study. We've ensured participant consistency, justified intervention activities, conducted a pilot test, broadened interview participant selection criteria, acknowledged statistical method limitations, and provided clear practical implications along with suggestions for future research. Your constructive feedback has been immensely valuable throughout this process.

**Reviewer #3:** All comments have been addressed

**Our response:** Thank you.

2. Is the manuscript technically sound, and do the data support the conclusions?

The manuscript must describe a technically sound piece of scientific research with data that supports the conclusions. Experiments must have been conducted rigorously, with appropriate controls, replication, and sample sizes. The conclusions must be drawn appropriately based on the data presented.

**Reviewer #2:** Partly

**Our response:**

Thank you for your feedback. We are pleased to hear that you found the manuscript to be partly technically sound. We have made every effort to ensure the rigor of our research, including appropriate controls, replication, and sample sizes. The data robustly support the conclusions drawn in the manuscript. Once again, thank you for your insights. We have taken them into consideration in further strengthening our study.

**Reviewer #3:** Yes

**Our response:** Thank you

3. Has the statistical analysis been performed appropriately and rigorously?

**Reviewer #2:** Yes

**Our response**: Thank you

**Reviewer #3:** Yes

**Our response:** Thank you

4. Have the authors made all data underlying the findings in their manuscript fully available?

The [PLOS Data policy](http://www.plosone.org/static/policies.action#sharing) requires authors to make all data underlying the findings described in their manuscript fully available without restriction, with rare exception (please refer to the Data Availability Statement in the manuscript PDF file). The data should be provided as part of the manuscript or its supporting information, or deposited to a public repository. For example, in addition to summary statistics, the data points behind means, medians and variance measures should be available. If there are restrictions on publicly sharing data—e.g. participant privacy or use of data from a third party—those must be specified.

**Reviewer #2:** Yes

**Our response:** Thank you

**Reviewer #3:** Yes

**Our response:** Thank you

5. Is the manuscript presented in an intelligible fashion and written in standard English?

PLOS ONE does not copyedit accepted manuscripts, so the language in submitted articles must be clear, correct, and unambiguous. Any typographical or grammatical errors should be corrected at revision, so please note any specific errors here.

**Reviewer #2:** Yes

**Our response:** Thank you

**Reviewer #3:** Yes

**Our response:** Thank you

6. Review Comments to the Author

Please use the space provided to explain your answers to the questions above. You may also include additional comments for the author, including concerns about dual publication, research ethics, or publication ethics. (Please upload your review as an attachment if it exceeds 20,000 characters)

**Reviewer #2:** The authors have not respond any comment from reviewer 2 (me) in the present author response letter.

**Our response:** We apologize for the oversight in not addressing your comments directly in the author response letter. We acknowledge the importance of addressing all reviewers' feedback comprehensively. Rest assured, we have carefully reviewed your comments and have implemented necessary revisions in the manuscript to address your concerns. Your input is highly valued, and we appreciate the opportunity to improve our work.

**Reviewer #3:** (No Response)

**Our response:** Thank you.

7. PLOS authors have the option to publish the peer review history of their article ([what does this mean?](https://journals.plos.org/plosone/s/editorial-and-peer-review-process#loc-peer-review-history)). If published, this will include your full peer review and any attached files.


If you choose “no”, your identity will remain anonymous but your review may still be made public.


**Do you want your identity to be public for this peer review?** For information about this choice, including consent withdrawal, please see our [Privacy Policy](https://www.plos.org/privacy-policy).

**Reviewer #2:** No

Reviewer #3: No
